# Combining network pharmacology and *in vitro* and *in vivo* experiments to study the mechanism of Keluoxin in the treatment of radiation nephropathy^†^

**DOI:** 10.1093/jrr/rrad050

**Published:** 2023-07-10

**Authors:** Lin Deng, Shaoqing Wang, Xingli Leng, Peng Yao, Cuicui Li, Yang Zheng

**Affiliations:** Nephrology Department of The Second Affiliated Hospital of Chengdu Medical College, China National Nuclear Corporation 416 Hospital, Chengdu, China, No. 4, North Section 4, Second Ring Road, Chengdu 610057, China; Nephrology Department of The Second Affiliated Hospital of Chengdu Medical College, China National Nuclear Corporation 416 Hospital, Chengdu, China, No. 4, North Section 4, Second Ring Road, Chengdu 610057, China; Nephrology Department of The Second Affiliated Hospital of Chengdu Medical College, China National Nuclear Corporation 416 Hospital, Chengdu, China, No. 4, North Section 4, Second Ring Road, Chengdu 610057, China; Nephrology Department of The Second Affiliated Hospital of Chengdu Medical College, China National Nuclear Corporation 416 Hospital, Chengdu, China, No. 4, North Section 4, Second Ring Road, Chengdu 610057, China; Physical Examination Center of General Hospital of Western Warzone, China, No. 270 Tianhui Road, Rongdu Avenue, Chengdu, Sichuan Province 610083, China; Nephrology Department of The Second Affiliated Hospital of Chengdu Medical College, China National Nuclear Corporation 416 Hospital, Chengdu, China, No. 4, North Section 4, Second Ring Road, Chengdu 610057, China

**Keywords:** radiation nephropathy, network pharmacology, traditional Chinese medicine, oxidative stress, pathogenic mechanism

## Abstract

Radiation nephropathy refers to kidney damage caused by radiation therapy for malignant tumours. Currently, the pathogenesis is unclear and there is a lack of effective treatment methods. With the development of traditional Chinese medicine, the role of traditional Chinese medicine in the protection of radiation nephropathy is receiving increasing attention. Therefore, in this study, we used X-ray intraperitoneal irradiation to construct a mouse model of radiation nephropathy and studied the protective effect of traditional Chinese medicine Keluoxin on radiation nephropathy. We first analysed the potential targets and pathways of Keluoxin in the treatment of radiation nephropathy using network pharmacology methods, combined with *in vitro* and *in vivo* experimental verification, to study its potential mechanism. By searching the database, 136 components of Keluoxin were identified. A total of 333 intersectional targets related to radiation nephropathy were obtained. Among them, key targets include IL-6, TNF-α, HIF-1α, STAT1, STAT3, JAK1, JAK2, etc. *In in vivo* and *in vitro* experiments, we found that as the irradiation dose increased and time prolonged, kidney damage in mice gradually worsened in a time-dependent and dose-dependent manner. As the irradiation dose increases, the expression of pro-inflammatory factors Il-6, TNF-α, TGF-β increased. Compared with the irradiation group, the intervention of Keluoxin can reduce kidney damage caused by X-ray irradiation and reduce the expression of IL-6, TNF-α, TGF-β, STAT1, STAT3, JAK1, JAK2, etc. These results indicated that Keluoxin can alleviate kidney damage caused by X-ray irradiation, possibly by regulating the JAK/STAT signalling pathway, reducing inflammation levels and oxidative stress damage.

## INTRODUCTION

Radiotherapy is an important tool in the treatment of malignant tumours. The latest data show that >19 million new cases of cancer were diagnosed in 2020, of which ~9 million resulted in death. It is predicted that the number of cancer patients will exceed 28.4 million by 2040 [1]. The role and status of radiotherapy in cancer treatment are becoming increasingly prominent. Approximately, 70% of cancer patients need radiotherapy during cancer treatment, and ~40% of cancers can be cured by radiotherapy. The cure rate of radiotherapy is second only to that of surgery. The rapid development of radiotherapy has brought good news to countless tumour patients and prolonged their survival. Furthermore, during radiation killing of tumour cells, large doses of radiation exposure also damage surrounding tissues and organs. The damage caused by radiotherapy is divided into early effects and late effects [2]. Early effects usually appear 1–2 weeks after radiotherapy and include skin mucosal lesions (such as erythema, dryness and rupture, mucositis) and gastrointestinal symptoms (such as nausea, vomiting and diarrhoea). Late effects may appear months or years after treatment and include radiation-induced fibrosis, atrophy, vascular damage, nerve damage or the induction of a second tumour. Early effects are generally transient and usually stabilize or disappear after a few weeks, while late effects are irreversible and may get progressively worse. The kidney is a late-onset radiosensitive organ [3]. Radiation nephropathy occurs mainly due to radiotherapy for abdominal or pelvic tumours. Radiation nephropathy is an important issue that cannot be ignored, as it not only increases the financial burdens of patients and significantly decreases their quality of life, but also limits the radiation doses available for tumour control. With advances in medical technology, cancer survivors are increasing. Statistical analysis of the survival of cancer patients in the USA in 2022 showed that more than half of cancer patients can survive for 10 years or more [4]. With the increase of cancer patients and the extension of survival time, preventing and reducing the late side effects of radiation therapy are becoming increasingly imperative.

The kidney is a late-onset radiosensitive organ. The accumulation of radiation doses (single dose of 2 Gy) greater than 20 Gy will cause kidney damage [5, 6], which manifests as oedema, hypertension, renal hypofunction and different degrees of anaemia. Large doses of radiation will damage most or all components of the kidney, starting with glomerular damage, which affects endothelial cells and thylakoid cells, and gradually developing into glomerular fibrosis. Subsequently, tubular injury occurs, which includes tubular lysis, tubular interstitial scar formation, lumen enlargement and the release of inflammatory mediators. Yan Zhou used a linear accelerator to irradiate rats to study radiation-induced kidney injury [7]. The results showed that after different doses of radiation, glomerular lesions appeared earlier than renal tubular and renal interstitial lesions and gradually worsened with time. The time until the development of glomerular lesions was shorter in response to 15 Gy of radiation. Another study showed that 8 Gy of γ-radiation to the kidney could induce glomerular atrophy, tubular dilatation and nuclear consolidation [8].

The pathogenic mechanism of radiation nephropathy is still unclear, and effective treatment measures are lacking. The main drugs used to treat radiation damage, such as sulphur-containing radiation protectants, have obvious toxic side effects and exert antiradiation effects only when toxic doses are used [9]. Chinese medicine has greater potential in the treatment of radiation nephropathy. Many herbal ingredients have been shown to inhibit radiation damage. Total flavonoids of Astragalus can protect the spleen and thymus from radiation-induced damage, protect the haematopoietic system, reduce the decrease in peripheral blood cells and improve immunity [10]. Astragalus methyloside can reduce hepatocyte death due to gamma radiation exposure and decrease intracellular Reactive oxygen species(ROS)levels [11]. According to traditional Chinese medicine, radiation is hot and poisonous. Radiation rays invading the body can cause damage to the skin, mucous membranes, and organs, leading to insufficient Qi and blood, and consuming the body’s vital energy [12]. According to Professor Zhou Zhongying, a master of Chinese medicine, the pathogenesis of radiation injury is a combination of heat and stasis, and injury to both Qi and yin [13]. In addition, the prevention and treatment principles of promoting blood circulation, removing blood stasis, nourishing Yin and replenishing Qi have been proposed. The pathogenesis of radiation injury in Chinese medicine is based on deficiencies in Qi, blood, Yin and fluids and stasis and blood blockage. Therefore, the treatment is based on consolidating the root and cultivating the elements, which benefits Qi and fluid and invigorates blood to resolve stasis [14]. Keluoxin consists of Astragalus membranaceus, Radix pseudostellariae, Ligustrum lucidum, Chinese wolfberry, Chinese rhubarb and leech. Astragalus membranaceus and Radix pseudostellariae are the monarch medicines, which are combined to enhance the effects of nourishing Yin and tonifying Qi. Ligustrum lucidum and Chinese wolfberry are used as official medicines, and their combined use enhances the nourishing effect on the liver and kidneys. Rhubarb and Leech are adjuvant medicines. In clinical practice, Keluoxin is mainly used for diabetic nephropathy with Qi and Yin deficiency syndrome, which is in line with the Chinese medical pathology of radiation nephropathy. Therefore, this study conducted a more in-depth study on Keluoxin. However, the bioactive components and pharmacological mechanism of action of Keluoxin are not yet clear.

With the rapid development of bioinformatics, network pharmacology has become a powerful tool for exploring TCM [15]. Based on systems biology, multidirectional pharmacology and high-throughput analysis, network pharmacology can thoroughly explain the complex relationships between drugs and diseases by constructing biological networks and visualizing networks of potential active ingredients, targets, signalling pathways and diseases [16]. Therefore, network pharmacology can effectively explore the multiple components, targets and pathways of traditional Chinese medicine.

In this study, we analysed the bioactive components, targets of Keluoxin and targets and pathways of radioactive nephropathy by network pharmacology and constructed an active component-target-pathway-disease network. The molecular mechanism of Keluoxin in the treatment of radiation nephropathy was also verified by *in vivo* and *in vitro* experiments.

## MATERIALS AND METHODS

### Network pharmacology analysis

#### Potential target intersections of Keluoxin and radiation nephropathy

Six Chinese herbs in Keluoxin were used as keywords to search the TCM Systematic Pharmacology Database (TCMSP, http://tcmspw.com/tcmsp.php) and screen ingredients and targets [17]. This database can provide information on the composition and targets of herbal medicines. In this study, the parameters oral bioavailability (OB) and drug-like (DL) were used to screen the active ingredients and targets of the drugs [18]. DL is a qualitative concept used in drug design to estimate the ‘drug-Like’ quality of the intended compound. The DL index can be used to optimize pharmacokinetics and drug properties, such as solubility and chemical stability [19]. In general, we assigned an OB value ≥30% or DL value ≥0.18 as criteria for identifying potential active ingredients [20]. Leech was used as a keyword to search in TCMSP, but its chemical components were not obtained. Thirty-four kinds of leech chemical constituents were obtained from related literature [21–23], and the online data platform pubChem (https://pubchem.ncbi.nlm.nih.gov/) was used to obtain the SDF format of each component and record it in Swiss target ADME (http://www.swissadme.ch/index.php). A total of 18 active ingredients in leech were finally obtained by screening ingredients with good oral availability and DL properties.

Radiation nephropathy was used as a keyword to search the Disgenet database (https://www.disgenet.org/), OMIM database (https://omim.org/) and Gene Cards database (https://www.genecards. org/) to identify targets related to radiation nephropathy. After removing the duplicate targets, the final targets of radiation nephropathy were obtained. The gene datasets obtained to screen radioactive nephropathy-associated targets and Keluoxin -associated targets were imported into the online Venn diagram (https://bioinfogp.cnb.csic.es/tools/venny/index.html) to determine the intersecting genes. The STRING database was used to determine the interaction relationships between the target proteins [24].

#### Pathway and functional enrichment analysis

GO functional enrichment analysis and KEGG pathway enrichment analysis are common analytical methods to elucidate the roles of target proteins of compounds in gene functions and signalling pathways [25]. In this study, the key target proteins shared by Keluoxin and radiation nephropathy were functionally annotated and enriched to investigate the key targets and related signalling pathways of Keluoxin in the treatment of radiation nephropathy and investigate their mechanisms of action.

#### Network construction and analysis

Cytoscape 3.8.0 software was used for biological network visualization and data integration analysis. The software was used to build disease-component-target map networks, protein–protein interaction (PPI) networks, and compound-target-pathway networks.

### Experimental verification

#### Reagents and materials

Eight-week-old C57BL/6 mice were purchased from Da Shuo Technology Co, Ltd (Chengdu, China) for this experiment, and all animal procedures in this study were approved by the Ethics Committee of The Second Affiliated Hospital of Chengdu Medical College, China National Nuclear Corporation 416 Hospital. Keluoxin was purchased from Kanghong Pharmaceutical Group of Chengdu. Foetal bovine serum, phosphate buffer solution (PBS), DMEM, a penicillin and streptomycin mixture, Cell Counting Kit 8 (CCK-8) kits and lactate dehydrogenase (LDH) kits were purchased from Beijing Solabo Technology Co, Ltd. creatinine (CR), blood urea nitrogen (BUN), IL-6, TNF-α, TGF-β and IFN-γ kits were purchased from Shanghai Biyuntian Technology Co, Ltd. Trypsin, JAK1, JAK2, STAT1, STAT3 and HIF-1α antibodies were purchased from Proteintech (Wuhan, China).

#### Preparation of serum containing Keluoxin

Male SD rats weighing 200–250 g were randomly divided into two groups (*n* = 12): the drug-containing serum group and the blank serum group. After 1 week of acclimatization and feeding, the rats in the drug-containing serum group were given Keluoxin (3.78 mg/g/day) by gavage for 3 days, and the blank serum group was given the same volume of saline. Blood was collected via the abdominal aorta on the fourth day and centrifuged for 10 min (4°C, 3000 r/min) after standing for 4 h. The serum was sterilized in a water bath at 56°C for 30 min and by filtration with 0.22 μm microfilm pores, after which it was mixed and divided into 1.5 ml EP tubes and stored at −80°C.

#### Cell line and culture

The mouse renal tubular epithelial (TCMK-1) cell line was purchased from GuangZhou Jennio Biotech Co, Ltd. The cells were incubated in DMEM supplemented with 10% FBS, 100 U/ml penicillin and 100 μl/ml streptomycin. Cells at ~80% confluence were digested with 0.25% trypsin–EDTA and seeded in 96-well plates or petri dishes. After the cells adhered to the surface, different doses of X-ray irradiation were administered. CCK-8 or Lactic dehydrogenase (LDH) kits were used to detect cell viability and mortality.

#### C‌CK-8 cytotoxicity assay

The cytotoxicity of X-ray radiation was determined by CCK-8 assays. The cells were seeded onto 96-well plates and cultured until they adhered completely. Then, the cells were irradiated with different doses of X-ray radiation (0, 2.5, 5, 7.5, 10, 12.5 and 15 Gy). Four hours before irradiation, the medium was replaced with DMEM containing 6% serum containing the drug, and the culture was continued for 12, 24 and 48 h after irradiation. Then, 10 μl of CCK-8 was added and incubated for another 4 h. The absorbance was recorded at 450 nm, and the experiments were performed in parallel in triplicate.

#### Lactic dehydrogenase (LDH) assay

The cytotoxicity of X-ray radiation was determined by LDH assays. The cells were seeded onto 96-well plates and cultured until they adhered completely. Blank control wells and maximum enzymatic activity wells were set up. The original culture medium was aspirated, the cells were washed one to two times with PBS and serum-free medium was added. LDH assay working solution was added and incubated for 30 min. The absorbance was recorded at 450 nm, and the experiments were performed in parallel in triplicate.

#### Experimental groups and radiation method

To construct and validate a radioactive nephropathy model, 24 mice were randomly divided into four groups (*n* = 6): CON group (dose = 0 Gy), RN 1 group (dose = 8 Gy), RN 2 group (dose = 10 Gy) and RN3 group (dose = 12 Gy). All mice of the RN group were given X-ray intraperitoneal irradiation at a dose rate of 300ccy/min and at a distance of 100cm from the skin. Cover other parts of the body with lead plates. All mice were continued to be fed for 3–5 months. They were then executed in batches, and mouse serum and kidney tissue were collected for renal function testing.

To study the protective effect of Keluoxin on radioactive nephropathy, 18 mice were randomly divided into three groups: the control group (CON), radiation nephropathy group (RN, 12 Gy X-ray irradiation) and Keluoxin group (KLX, 12 Gy X-ray irradiation+ Keluoxin, 900 mg/kg, po), with six mice in each group. The control group was treated with an equal volume of saline, which was administered gavage for 4 months after irradiation. After 4 months, all mice were fasted for 12 h and then sacrificed, and serum samples and kidney tissue specimens were prepared for further experiments.

#### Enzyme-linked immunosorbent assay (ELISA)

The mice were intraperitoneally anaesthetized by chloral hydrate (10%), and blood samples were collected from the eyeball on the 120th day after irradiation. The blood samples were kept at room temperature for 12 h and then centrifuged for 15 min (4°C, 3500 r/min). The supernatants were collected and stored at −80°C until use. According to the manufacturer’s protocols, the levels of Malondialdehyde (MDA), Reduced glutathione (GSH), TGF-β, IL-6, TNF-α and γ-INF were measured by commercial ELISA kits.

#### Renal pathological examination

Unilateral renal tissue was fixed with paraformaldehyde (4%) (Sigma–Aldrich) and then dehydrated in ethanol and embedded in paraffin. The 4-μm paraffin sections were prepared for haematoxylin-eosin (H&E) and Masson’s trichrome staining. The remaining kidney tissues were immediately snap frozen in liquid nitrogen and then transferred to a −80°C freezer for storage and subsequent western blot analysis.

#### Immunohistochemistry

Paraffin-embedded kidney sections were separated in xylene and rehydrated through a descending ethanol gradient. The sections were immersed in EDTA antigen repair buffer (pH 8.0) and then boiled in a microwave oven at high power for 10 min. After naturally cooling, the sections were washed three times in PBS (pH 7.4) for 3 min each. The sections were incubated in 3% H_2_O_2_ for 25 min at room temperature in the dark, followed by three washes in PBS (pH 7.4) for 5 min each. The sections were incubated overnight at 4°C in primary antibodies and washed three times in PBS (pH 7.4) for 5 min each. The primary antibodies used were anti-collagen I, anti-collagen III and anti-α-SMA. Appropriate secondary antibodies (anti-atrial or anti-rabbit) were added to the slides and incubated for 20 min at 37°C, followed by four washes in PBS for 3 min each. The sections were stained with 3,3-diaminobenzidine (DAB), counterstained with haematoxylin and sealed with neutral gel. Image acquisition was performed using light microscopy.

#### Western blotting

The kidney tissue was lysed in RIPA buffer (containing PMSF and a phosphatase inhibitor) for 30 min on ice. The total protein concentration was determined by a BCA protein assay kit. Equal amounts of protein (30 μg) were separated by 10% SDS–PAGE and then electrophoretically transferred onto PVDF membranes. The membranes were blocked with 5% fat-free milk in TBST buffer for 2 h at room temperature and then incubated at 4°C overnight with the following primary antibodies: anti-JAK1 (1:1000), anti-JAK2 (1:1000), anti-STAT1 (1:1000) and anti-STAT3 (1:1000). Then, the membranes were incubated with HRP-conjugated anti-rabbit/mouse IgG. The blots were imaged with an enhanced chemiluminescence (ECL) system.

### Statistical analysis

All data are expressed as the mean ± standard deviation ($\overline{\mathrm{X}}$ ± S). GraphPad Prism 9 software was used to determine statistically significant differences. The difference between the means was considered statistically significant when *P* < 0.05. *P* ≤ 0.01 was considered significantly different.

## RESULTS

### Identification of bioactive components in Keluoxin

By searching related databases and the literature, 20 components of Scutellariae, 13 components of Ligustrum, 16 components of Rhubarb, 8 components of Radix pseudostellariae, 34 components of leech and 45 components of Wolfberry were identified. The bioactive components are shown in Table 1, and the topological parameters of the top 20 active compounds in Keluoxin are shown in Table 2.

**Table 1:** The number of active components and corresponding target genes of Keluoxin

Drugs	Components	Target genes
Astragalus membranaceus	20	449
Ligustrum lucidum	13	219
Chinese rhubarb	16	191
Radix pseudostellariae	8	303
Chinese wolfberry	45	511
Leech	18	158

**Table 2:** Topological parameters of the top 20 active compounds in Keluoxin

Serial number	Active compounds	DC	BC	CC
1	Sitosterol alpha1	89	0.05040081	0.41006524
2	Mandenol	78	0.05936058	0.39675383
3	24-Ethylcholest-22-enol	70	0.05943618	0.39747064
4	24-Methyl-31-norlanost-9(11)-enol	65	0.00891	0.37068239
5	(3R)-3-(2-hydroxy-3,4-dimethoxyphenyl)chroman-7-ol	56	0.0587282	0.3783319
6	Jaranol	56	0.02401037	0.38834951
7	Myristoleic acid	56	0.00049	0.38834951
8	(6aR,11aR)-9,10-dimethoxy-6a,11a-dihydro-6H-benzofurano[3,2-c]chromen-3-ol	55	0.05900167	0.38698329
9	Quercetin	55	0.02383864	0.39180766
10	Isorhamnetin	55	0.01820076	0.38495188
11	Kaempferol	54	0.02214749	0.38972542
12	14-methyl Pentadecanoic Acid methyl ester	50	0.04522032	0.38095238
13	Ethyl linolenate	47	0.04402439	0.38029386
14	3,9-Di-O-methylnissolin	46	0.04571671	0.3783319
15	Tridecylic acid	46	0.0154904	0.37510656
16	1-Monolinolein	44	0.02918105	0.37256562
17	Atropine	44	0.05953304	0.37383178
18	Syringaresinol diglucoside_qt	44	0.025951	0.38161318
19	12-Methyl Myristic Acid methyl ester	45	0.03726736	0.37130802
20	Lauric acid	44	0.0220047	0.37383178

DC = degree centrality, BC = between centrality, CC = closeness of centrality.

### Compound-target-pathway network construction

**Fig. 1:**
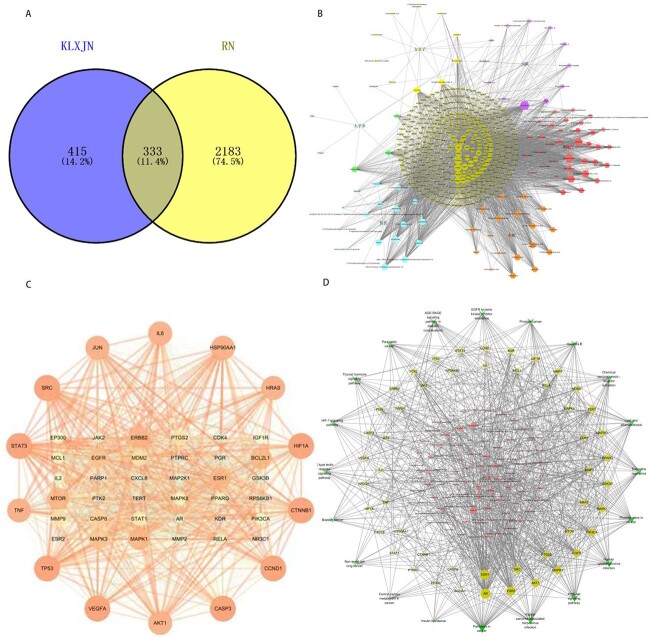
The network of Keluoxin in the treatment of radiation nephropathy. (A) Venn diagram of the intersecting targets of Keluoxin and radiation nephropathy. (B) The compound-target network of Keluoxin. (C) The interaction network of 50 key target proteins. (D) The compound-target-pathway network.

Radiation nephropathy was used as a keyword to identify disease targets related to radiation nephropathy in the Disgenet database, OMIM database and Gene Cards database. The gene datasets obtained from the screening of radiation nephropathy-related targets and Keluoxin were imported into the online Venn diagram, and a total of 333 intersecting targets were obtained, as shown in Fig. 1A. The relevant components and targets of Keluoxin and radiation nephropathy were introduced into Cytoscape 3.7.2 to obtain a diagram of traditional Chinese medicine component targets (Fig. 1B).

### Protein-protein interaction network analysis

**Fig. 2:**
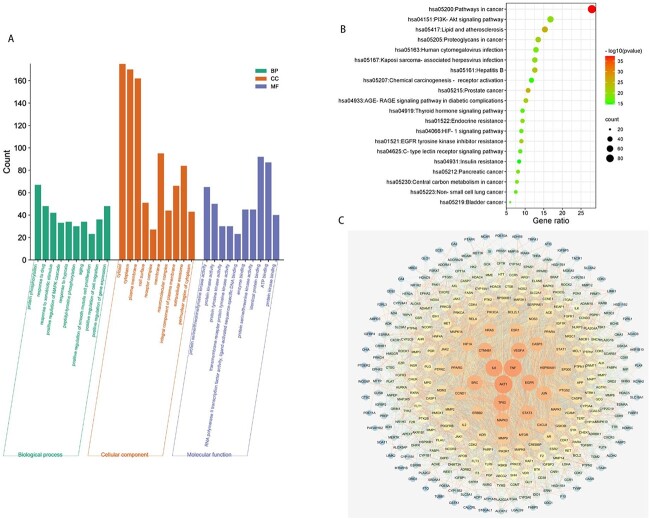
PPI network analysis and enrichment analysis. (A) Gene Ontology (GO) enrichment analysis of key targets. (B) KEGG pathway enrichment analysis of key targets. (C) PPI network of protein targets from the STRING database.

The targets of Keluoxin were mapped with the targets of radioactive nephropathy, and the mapping results were imported into the STRING database. Multiple proteins and *Homo sapiens* were used to construct a protein interaction network and obtain the *in vivo* reflection network of Keluoxin with radioactive nephropathy. A network graph containing 333 nodes and 6464 edges was obtained (Fig. 2C). Using the plug-in MCODE, the network was constructed by selecting ‘In whole network’, setting degree cut-off = 2, selecting ‘Haircut’ and setting node score cut-off = 0.2, K-core =2，Max. Depth = 100. The first 50 targets of the K-core were selected as the key targets (Fig. 1C).

### GO enrichment analysis

GO enrichment analysis of the 333 potential therapeutic targets was performed to identify the relevant biological functions by which Keluoxin protects against radiation injury. The top 10 significantly enriched terms with an increased number of involved targets in the biological process (BP), cellular component (CC) and molecular function categories are shown in Fig. 2A, and the results indicate that Keluoxin may regulate cell proliferation, apoptosis, migration and senescence and the response to hypoxia by adjusting protein serine/threonine kinase activity in the plasma membrane, extracellular region and cytosol to exert therapeutic effects against radiation injury.

### Pathway enrichment analysis

To explore the potential pathways by which Keluoxin protects against radiation injury, pathway enrichment analysis of the 167 potential therapeutic targets was performed. The top 20 significantly enriched pathways are shown in Fig. 2B. Among these potential pathways, cancer signalling pathways were the most prominently enriched according to the gene numbers. The HIF-1 signalling pathway, insulin resistance, lipid and atherosclerosis and PI3K-AKT signalling pathways were also included, which were related to inflammation, oxidative stress, hypoxia, vascular calcification and endocrine resistance. Because genes cannot exert their biological and pharmacological effects independently, a compound-target-pathway network was established based on the top 20 signalling pathways and the involved targets and compounds to further elucidate the molecular mechanism by which Keluoxin protects against radiation injury (Fig. 1D). After integrating drug target prediction, pathway and functional enrichment and network analyses, we identified TNF-α, IL-6, STAT1, STAT3 and JAK2 as relatively highly relevant targets in inflammation, hypoxia and oxidative stress. Thus, we hypothesized that Keluoxin can regulate inflammation, hypoxia and oxidative stress by targeting the TNF, IL-6, JAK/STAT signalling pathways to inhibit radiation damage.

### Experimental verification

#### Effect of Keluoxin on cytotoxicity after X-ray irradiation

**Fig. 3:**
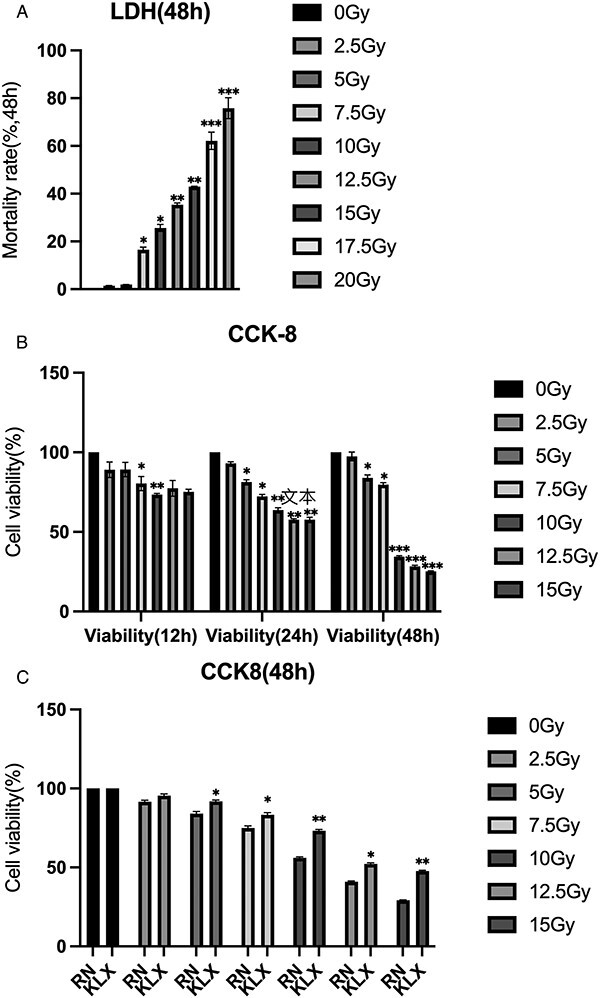
Effect of different doses of X-ray on the activity of TCMK-1 cells. (A) TCMK-1 cell mortality after different doses of X-ray irradiation. Measure cell mortality 48 h after X-ray irradiation. As the X-ray dose increases, the cell mortality rate gradually increases. (B) TCMK-1 cell viability at 12, 24 and 48 h after different doses of X-ray irradiation. Measure cell mortality 12, 24 and 48 h after X-ray irradiation. As the X-ray dose increases, the cell survival rate gradually decreases. The most significant decrease in cell viability was observed 48 h after 10 Gy X-ray irradiation. (C) Effect of medicated serum containing Keluoxin on TCMK-1 cell viability. As the X-ray dose increases, the cell survival rate gradually decreases and medicated serum-containing Keluoxin can inhibit this decline (^*^P < 0.05, ^*^^*^P < 0.01, ^*^^*^^*^P < 0.001).

The viability of TCMK-1 cells was inhibited dose- and time-dependently after exposure to different radiation doses of X-ray irradiation for 12, 24 and 48 h **(**Fig. 3A and B). Cell viability was significantly inhibited by 10 Gy X-ray irradiation for 48 h (*P* < 0.01). When 6% medicated serum was added to the cells for 48 h, cell viability was significantly increased, especially in response to 10 Gy X-ray irradiation (*P* < 0.01). Thus, Keluoxin can improve the survival rate and activity of TCMK-1 cells after X-ray irradiation (Fig. 3C).

#### Medicated serum containing Keluoxin reduces X-ray irradiation-induced oxidative stress levels

**Fig. 4:**
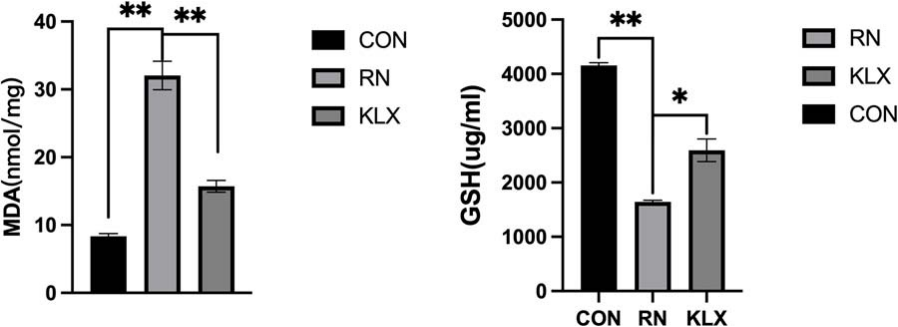
Effect of Keluoxin on lipid peroxidation of TCMK-1 after X-ray irradiation. (A, B) Effect of serum-containing Keluoxin on MDA and GSH levels after X-ray irradiation. After 10 Gy irradiation, the expression of MDA increased and the expression of GSH decreased in TCMK-1 cells, with statistical differences (all compared with CON groups). And medicated serum-containing Keluoxin can inhibit these changes.

To investigate the effect of medicated serum containing Keluoxin on oxidative stress in TCMK-1 cells, MDA and GSH levels were measured before and after irradiation. The radiation nephropathy group had higher MDA levels and lower GSH levels than the control group. GSH levels were significantly increased, and MDA levels were decreased after the treatment with Keluoxin-containing serum (Fig. 4).

#### Radiation nephropathy model validation

**Fig. 5:**
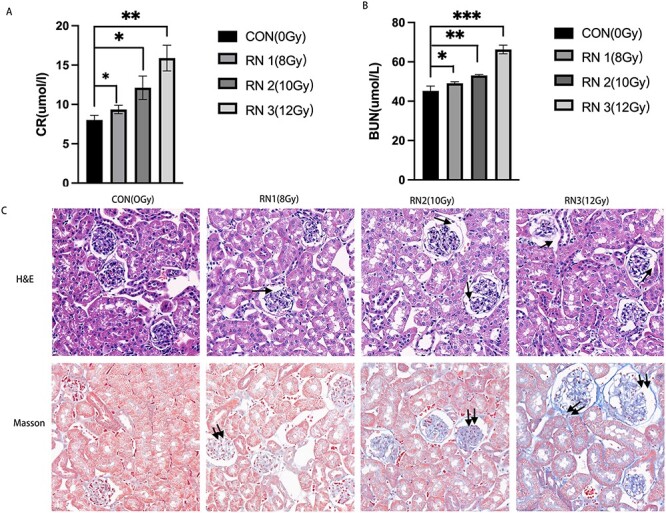
Renal function and kidney pathology in mice. (A) Effect of different irradiation doses on CR levels in mice. (B) Effect of different irradiation doses on BUN level in mice. (C) H&E and Masson staining images (40×) in the different irradiation dose groups (The arrow indicates glomerular atrophy and cystic dilation. Double arrows indicate changes in renal fibrosis.) ^*^P < 0.05, ^*^^*^P < 0.01, ^*^^*^^*^P < 0.001.

In this study, we used X-rays to irradiate the abdominal cavities of mice to construct a model of radiation nephropathy. Based on previous research results [26, 27], 8, 10 and 12 Gy were used for irradiation. We measured CR and BUN levels and the degree of renal injury in mice on the 120th day after irradiation. With increasing radiation doses, CR and BUN levels gradually increased (Fig. 5A and B and Table 3). H&E staining revealed that the kidneys exhibited diffuse inflammatory cell infiltration, deformation of glomerular vacuoles and dilatation of vesicles. Masson’s trichrome staining indicated that with increasing irradiation doses, there was a gradual increase in collagen fibre content (Fig. 5C).

**Table 3:** The levels of CR and BUN in each group of mice after X-ray irradiation

Group	CON (*n* = 6)		RN1 (*n* = 6)		RN2 (*n* = 6)		RN3 (*n* = 6)	
	mean ± SEM	*P-*value	mean ± SEM	*P-*value^*^	mean ± SEM	*P-*value^*^	mean ± SEM	*P-*value^*^
CR (μmol/l)	8.033 ± 0.569		9.356 ± 0.536	0.0463	12.120 ± 1.496	0.0282	15.892 ± 1.637	0.0014
BUN (μmol/l)	45.239 ± 2.489		49.099 ± 0.771	0.0208	53.068 ± 0.485	0.0100	66.297 ± 2.179	0.0001

^*^All compared with CON group. SEM = standard error of mean, RN1 = 8 Gy X-ray radiation, RN2 = 10 Gy X-ray radiation, RN3 = 12 Gy X-ray radiation.

#### Effect of Keluoxin on renal histopathology after X-ray irradiation

**Fig. 6:**
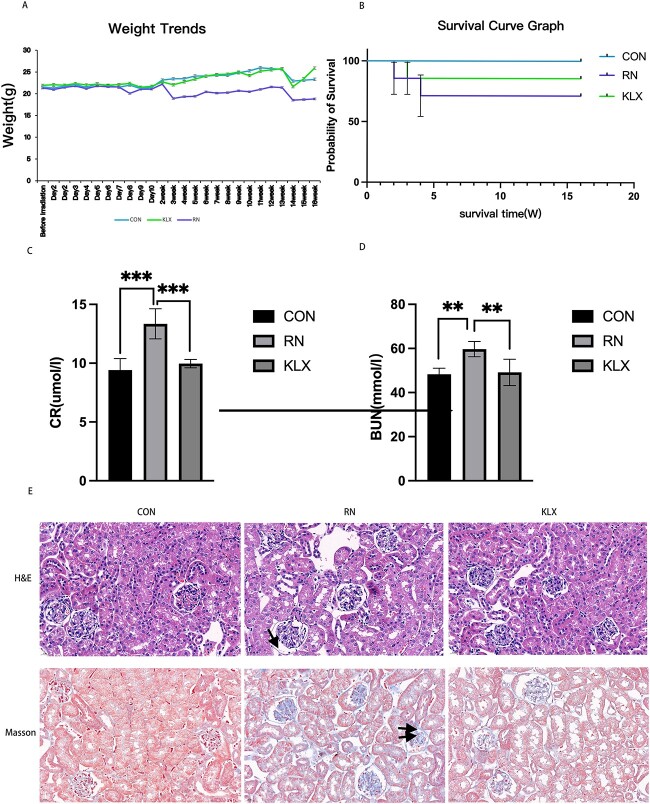
Effect of Keluoxin on renal function and pathology in radiation induced nephropathy. (A) Changes in mouse body weight in each group 16 weeks after irradiation. (B) Survival curves of mice in each group. (C, D) Effects of Keluoxin on CR and BUN levels in mice with radiation nephropathy. (E) H&E and Masson staining of kidney tissues (40×) (The arrow indicates glomerular atrophy and cystic dilation. Double arrows indicate changes in renal fibrosis.) ^*^^*^P < 0.01, ^*^^*^^*^P < 0.001.

To study the effect of Keluoxin on radiation nephropathy, different groups were established. The radiation nephropathy model was constructed using 12 Gy of X-ray irradiation, and the changes in body weight, survival rate, renal function and pathological damage were compared among the control group, radiation nephropathy group and Keluoxin group. The results showed that there was a certain degree of weight loss in mice 1–2 weeks after X-ray irradiation (Fig. 6A), which was considered to be related to radiation-induced enteritis [28, 29]. Compared with those in the radiation nephropathy group, CR and BUN levels were significantly decreased in the Keluoxin group (Fig. 6C and D and Table 4), and the degree of glomerular vacuolar degeneration and fibrosis was reduced (Fig. 6E).

**Table 4:** The levels of CR and BUN in each group of mice after X-ray irradiation

Group	CON (*n* = 6)		RN (*n* = 6)		KLX (*n* = 6)	
	mean ± SEM	*P-*value	mean ± SEM	*P-*value^*^	mean ± SEM	*P-*value^**^
CR (μmol/l)	9.418 ± 0.971		13.348 ± 1.284	0.0004	9.965 ± 1.357	0.0009
BUN (μmol/l)	48.331 ± 2.709		59.685 ± 3.465	0.0027	49.136 ± 5.955	0.0084

^*^All compared with CON group.

^**^All compared with RN group. SEM = standard error of mean, RN = 12 Gy X-ray radiation, KLX = 12 Gy X-ray radiation+ Keluoxin, 900 mg/kg)

#### Effect of Keluoxin on renal fibrosis after X-ray irradiation

**Fig. 7:**
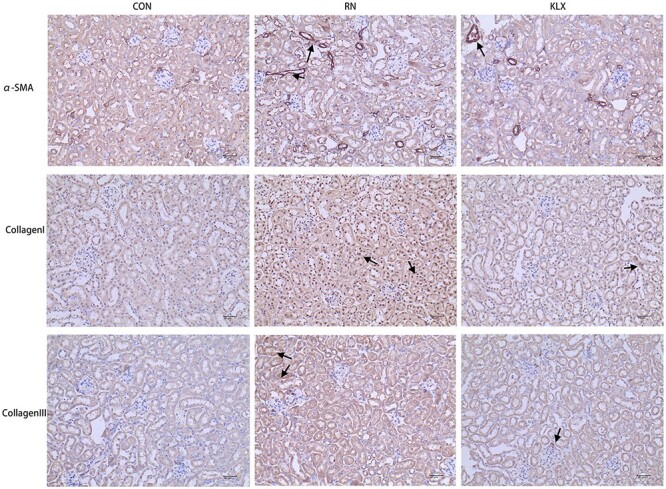
Immunohistochemical analysis of fibrosis indicators. The expression of collagen I, collagen III and α-SMA in mouse kidneys in the control group, radiation nephropathy group and Keluoxin group. The arrows indicate the positions of the fibrosis indicators.

To study the effect of Keluoxin on renal fibrosis after X-ray irradiation, we measured the expression of collagen I, collagen III and α-SMA. After X-ray irradiation, the expression of collagen I, collagen III and α-SMA in kidneys increased significantly. Keluoxin could inhibit the expression of these genes (Fig. 7).

#### Keluoxin reduces the inflammatory response, oxidative stress levels and renal fibrosis

**Fig. 8:**
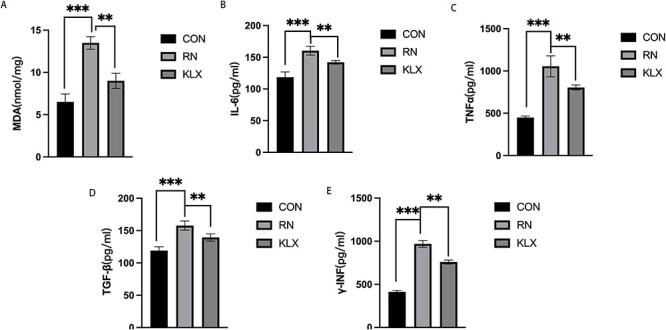
Effects of Keluoxin on inflammation and oxidative stress in radiation-induced nephropathy. Figures A-E correspond to the levels of MDA, IL-6, TNF-α, TGF-β and γ-IFN in the control, radiation and herbal medicine groups (^*^^*^P < 0.01, ^*^^*^^*^P < 0.001).

We examined IL-6, TNF-α, TGF-β, γ-IFN and MDA levels in the three groups. Compared with those in the control group, the levels of inflammatory indicators such as IL-6, TNF-a, r-IFN and MDA were significantly increased in the radiation nephropathy group (Fig. 8 and Table 5), and the fibrosis indicator TGF-β was also increased. Keluoxin treatment reduced inflammation, decreased the level of oxidative stress and reduced the degree of fibrosis caused by X-ray irradiation (Fig. 8).

**Table 5:** Expression levels of proinflammatory cytokines in different groups

Group	CON (*n* = 6)		RN (*n* = 6)		KLX (*n* = 6)	
	mean ± SEM	*P-*value	mean ± SEM	*P-*value^*^	mean ± SEM	*P-*value^**^
MDA (nmol/mg)	6.513 ± 0.940		13.489 ± 0.746	<0.001	9.002 ± 0.890	<0.01
IL-6 (pg/ml)	118.504 ± 8.484		160.372 ± 6.984	<0.001	142.133 ± 2.821	<0.01
TNF-α (pg/ml)	448.800 ± 18.480		1055.000 ± 124.700	<0.001	804.750 ± 29.503	0.003
TGF-β (pg/ml)	119.081 ± 5.967		157.745 ± 7.105	<0.001	139.353 ± 5.643	0.004
γ-IFN (pg/ml)	412.561 ± 17.424		969.333 ± 39.328	<0.001	748.871 ± 23.625	<0.01

^*^All compared with CON group.

^**^All compared with RN group. SEM: standard error of mean. TNF-α = tumour necrosis factor-α, IL-6 = interleukin-6, γ-INF = γ-interferon, TGF-β = transforming growth factor-β, MDA = malondialdehyde, RN = 12 Gy X-ray radiation, KLX = 12 Gy X-ray radiation+ Keluoxin, 900 mg/kg).

#### Keluoxin inhibits the JAK/STAT signalling pathways in mice with radiation nephropathy

**Fig. 9:**
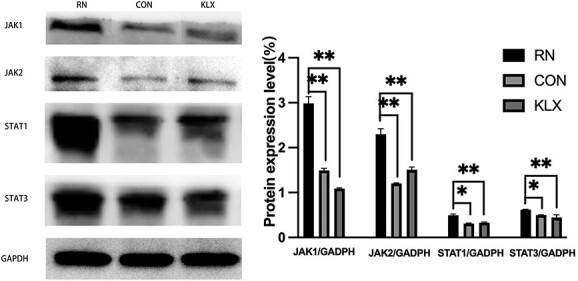
Effect of Keluoxin on the expression of JAK/STAT signalling pathway-related indicators. The expression of JAK1, JAK2, STAT1, STAT3 in in renal tissues in the different groups. The expression levels of JAK1, JAK2, STAT1, STAT3 were all significantly increased after radiation (all compared with CON groups), and all these significant changes after radiation were ameliorated by Keluoxin (^*^P < 0.05, ^*^^*^P < 0.01).

According to the component-target-pathway network, JAK/STAT may be the main pathways through which Keluoxin exerts its renoprotective effects against radiation nephropathy. As shown in Fig. 9, western blot analysis indicated that the expression of JAK/STAT was upregulated in the radiation nephropathy group compared with the control group (*P* < 0.01). After Keluoxin treatment for 4 months, the JAK/STAT signalling was significantly suppressed compared with those in the radiation nephropathy group.

## DISCUSSION

The pathogenesis of radiation nephropathy is not clear, and previous studies [27, 30] suggested that this condition may be related to inflammation, oxidative stress, apoptosis and other factors. According to the principles of TCM, radiation nephropathy belongs to fire-heat evil toxicity [12]. Radiation has both fire toxicity and heat toxicity [14]. The pathology of the disease involves excessive yang energy and hyperactive function, which increases the consumption of substances, thus injuring the yin and depleting the fluid. The pathogenesis is that the evil heat injures Yin, and Qi and blood are both deficient [13]. Professor Zhongying Zhou proposed the prevention and treatment principle of invigorating blood stasis, nourishing Yin and benefiting Qi. The herbal formula used in this study was formulated by Professor Lan Lin, a famous expert in Chinese and Western medicine, based on >40 years of clinical experience. It has been reported in the literature that Keluoxin can reduce NF-κB activation, decrease ROS and MDA levels in rats with oxidative damage, effectively reduce oxidative stress in hyperlipidaemic animals, activate blood circulation, resolve blood stasis, benefit Qi and nourish Yin, which can be used to treat radiological kidney injury in which blood stasis and heat are fighting against each other and Qi and Yin are injured.

The mechanism of radiation nephropathy is complex, and multiple targets and pathways are involved in the development of radiation nephropathy. Treatment of a single target cannot achieve the expected effect. Chinese herbal formulas have multiple components and multiple targets, which provides more possibilities for the treatment of radiation nephropathy. However, the complex composition of TCM formulas and the relationships between the components and the targets also pose great difficulties in exploring the potential mechanisms of TCM formulas. TCM exerts its pharmacological effects through the synergistic actions of multiple compounds with high pharmacological activity, and there are some toxic or low pharmacological activity compounds and impurities in TCM formulas that have no positive pharmacological effects in the treatment of diseases [31]. Therefore, when using network pharmacology to analyse the components of drugs, we generally set an OB value ≥30% or DL value ≥0.18 as the screening conditions to select the ingredients with high pharmacological activity for further study. In this study, after screening the database and related literature, 20 components of Scutellariae, 13 components of Ligustrum, 16 components of Rhubarb, 8 components of Radix pseudostellariae, 34 components of Leech and 45 components of Wolfberry were obtained. Among them, quercetin, mandenol, isorhamnetin and other components were the primary components. Isorhamnetin exerts anti-inflammatory and antioxidant effects by regulating the PI3K/AKT/PKB, NF-κB, MAPK and other signalling pathways [32].In addition to its powerful anti-inflammatory, antitumour and antioxidant effect, quercetin has insulin-modulating effects [33]. Isorhamnetin is widely recognized for its natural antioxidant effects; however, in addition to antioxidant effects, it can modulate autophagy and inhibit fibrosis [34, 35].

By constructing component-target-disease and ingredient-target-pathway-disease networks, we found that Keluoxin acts through various targets and pathways, among which the main targets included IL-6, TNF-α, HIF-1α, STAT3, AKT, JAK1, JAK2, STAT1 and STAT3, which are related to inflammation, oxidative stress, extracellular matrix production, angiogenesis and other functions. Oxidative stress is a central aspect of radiation nephropathy. Balalauli *et al*. [36] and Robbins *et al*. [37] found a dose-dependent increase in glomerular and tubular nuclear DNA oxidation after kidney irradiation, and these changes often occurred 4 weeks after irradiation and persisted until 24 weeks after irradiation, suggesting that chronic and persistent oxidative stress occurs in the kidney after irradiation. Oxidative stress exacerbates glomerular endothelial cell and tubular epithelial cell damage and increases the expression of inflammatory mediators such as PAI-1, MMP-2 and TGF-β, leading to the development of renal fibrosis. Combined with the network pharmacological analysis, Keluoxin may exert antifibrotic effects by regulating the JAK/STAT signalling pathways to reduce inflammatory responses and oxidative stress levels and inhibit extracellular matrix production. To further verify this result, we investigated the therapeutic effects of Keluoxin on a mouse radiation nephropathy model. The radiation nephropathy model was constructed using X-rays to irradiate the abdominal cavities of mice. To determine the optimal radiation dose for the model, we first irradiated TCMK1 cells with 0–20 Gy X-ray and observed the effects of different doses of X-ray on cell activity and mortality. We found that as the irradiation dose and irradiation time increased, the activity of TCMK1 gradually decreased and the mortality rate gradually increased. Among them, the most significant decrease in cell activity was observed 48 h after 10 Gy X-ray irradiation. Therefore, we set the irradiation doses for RN1, RN2 and RN3 groups to be 8, 10 and 12 Gy, respectively. There was significant weight loss in the mice 1–2 weeks after radiation treatment, which may be related to radiation-induced enteritis. X-ray irradiation with 12 Gy induced fibrotic changes after 3 months, while 8–10 Gy irradiation induced a large number of inflammatory cell infiltrates and significant glomerular vacuolar degeneration with milder fibrotic changes after 3 months, indicating that renal function damage is dose-and time-dependent, which is similar to the results of previous studies [2]. The pathological features of radiation nephropathy include inflammatory infiltration, glomerular vacuolar degeneration, tubular dilatation and excessive extracellular matrix deposition. Inflammation is an early response to kidney injury, and chronic, persistent inflammation and oxidative stimulation exacerbate glomerular endothelial cell and tubular epithelial cell injury, increase ROS and MDA production and increase the expression of inflammatory mediators such as IL-1, IL-6 and TNF, gradually exacerbating renal fibrosis [38]. When the kidney is subjected to persistent injury, renal tubular epithelial cells are converted to myofibroblasts through a BP called the epithelial mesenchymal transition, which eventually results in fibrosis [39]. Our study showed that Keluoxin inhibited inflammatory infiltration in the kidney, improved glomerular vacuolar degeneration, reduced tubular dilatation and decreased serum levels of IL-1, IL-6 and TNF-α.

The JAK/STAT signaling pathway is the most dominant pathway regulating oxidative stress. When cytokines bind to receptors outside the cell membrane, the cytokine receptors are activated and transmit the signal to JAK kinase, which phosphorylates and further phosphorylates the downstream molecule STAT, which can enter the nucleus and act as part of the transcription factor complex to control the transcription of cellular genes [40]. Oxidative stress can exacerbate radiation nephropathy by increasing the renal production of components related to oxidative damage, such as ROS and MDA, which induces the phosphorylation of JAK1 in the JAK/STAT pathway, and JAK1 activation rapidly activates and phosphorylates STAT3 (p-STAT3). p-STAT3 in turn increases the expression of oxidative factors and exacerbates radiation nephropathy [41]. Our study showed that Keluoxin could regulate the JAK/STAT pathway, thus reducing radiation-induced inflammation and oxidative stress-induced damage and protecting against radiation nephropathy.

## CONCLUSION

In this study, the molecular mechanism of Keluoxin in the treatment of radiation nephropathy was investigated by using network pharmacology combined with *in vitro* and *in vivo* experimental validation. Central targets, such as IL-6, TNF-α, HIF-1α, STAT1, STAT3, JAK1 and JAK2, and the JAK/STAT pathways were identified by network pharmacology analysis and validated by *in vitro* and *in vivo* experiments, confirming that Keluoxin could reduce the inflammatory response in radiation nephropathy by regulating the JAK/STAT pathways, thereby reducing the level of oxidative stress and renal fibrosis and playing a role in renal protection.

## CONFLICT OF INTEREST

This manuscript is original, has not already been published, and is not currently under consideration by another journal. All the authors and the institutions where the work has carried out have approved submission of this manuscript. All animal procedures in this study were approved by the Ethics Committee of Chengdu Medical College Second Affiliated Hospital The Second Affiliated Hospital of Chengdu Medical College, China National Nuclear Corporation 416 Hospital.

## FUNDING

This work was supported by the Key Projects of the Nuclear Medicine Science and Technology Innovation of CNNC in 2021 (ZHYLZD2021005), the Scientific Research Project of Traditional Chinese Medicine of Sichuan Administration of Traditional Chinese Medicine (grant number 2021MS517) and the Medical Research Project of Medical Health Public Welfare Foundation of Beijing (grant number YWJKJJHKYJJ-KH19007).

## DATA AVAILABILITY

The datasets used and/or analyzed during the current study are available from the corresponding author on reasonable request.
